# Development of the Positive Emotions Program for Schizophrenia: An Intervention to Improve Pleasure and Motivation in Schizophrenia

**DOI:** 10.3389/fpsyt.2016.00013

**Published:** 2016-02-17

**Authors:** Alexandra Nguyen, Laurent Frobert, Iannis McCluskey, Philippe Golay, Charles Bonsack, Jérôme Favrod

**Affiliations:** ^1^School of Nursing Science La Source, University of Applied Sciences and Arts of Western Switzerland, Lausanne, Switzerland; ^2^Social Psychiatry Section, Community Psychiatry Service, Department of Psychiatry, University Hospital Center, Lausanne, Switzerland

**Keywords:** schizophrenia, anhedonia, apathy, pleasure, motivation, psychosocial interventions

## Abstract

**Objectives:**

The efficacy of drug-based treatments and psychological interventions on the primary negative symptoms of schizophrenia remains limited. Recent literature has distinguished negative symptoms associated with a diminished capacity to experience, from those associated with a limited capacity for expression. The positive emotions program for schizophrenia (PEPS) is a new method that specifically aims to reduce the syndrome of a diminished capacity to experience.

**Methods:**

The intervention’s vital ingredients were identified through a literature review of emotion in schizophrenia and positive psychology. The program has been beta-tested on various groups of health-care professionals.

**Results:**

A detailed description of the final version of PEPS is presented here. The French version of the program is freely downloadable.

**Conclusion:**

PEPS is a specific, short, easy to use, group-based intervention to improve pleasure, and motivation in schizophrenia. It was built considering a recovery-oriented approach to schizophrenia.

## Introduction

Negative symptoms have long been recognized as a central feature of the phenomenology of schizophrenia, dating back to early descriptions by Kraepelin and Bleuler (1). They negatively affect patients’ longitudinal social, occupational, and functional outcomes, as well their long-term recovery (2–5). Whereas positive symptoms (hallucinations, delusions) reflect an excess or distortion of normal functions, and negative symptoms (blunted affect, alogia, apathy–avolition, anhedonia, inattentiveness) represent the absence or reduction of normal emotions and behaviors. Negative symptoms are classified as primary or secondary. Primary negative symptoms comprise the core features intrinsic to schizophrenia itself. Secondary negative symptoms are transient; they are attributable and temporally related to the effects of such factors as unrelieved positive symptoms, depression, the adverse effects of antipsychotic drugs (akinesia), or the social isolation imposed by the stigma of schizophrenia. Primary and secondary negative symptoms may be similar in clinical expression, despite their contrasting etiologies (6). Often, secondary negative symptoms diminish with the resolution of their causative factors.

### Limitations of Current Techniques

The efficacy of drug-based treatments and psychological interventions on primary negative symptoms remains limited, however (7–9). Fusar-Poli et al. (9), in their meta-analysis of 6,503 patients in the treatment arm and 5,815 patients in the placebo arm, showed that most treatments reduced negative symptoms at follow-up relative to placebo: second-generation antipsychotics, antidepressants, combinations of pharmacological agents, glutamatergic medications, and psychological interventions. However, none of the treatments used reached the threshold for clinically significant improvement as measured by clinicians using the Clinical Global Impression Severity Scale. The mean percentage change in treatment groups was 16.1%, whereas the control group changed by an average 7.9%. Improvements by treatment group, compared with the control group, varied between 4.8% for first-generation antipsychotics and 12.7% for psychological treatments. There is a clear clinical need for developing treatments for negative symptoms. The lack of clinically meaningful efficacy of drug or psychological treatments is in line with clinicians’ practical experiences (7, 9). The existing psychological treatments have limitations (10). Family interventions to reduce negative symptoms appear promising when combined with other interventions. However, patients who have family members willing to be involved in treatment may merely represent a subgroup of people with schizophrenia. Furthermore, families who are more willing to participate in psychosocial interventions may provide patient’s with greater support anyway and thus may not represent the broader population. Psychosocial interventions, such as cognitive behavior therapy (CBT), social skills training (SST), mindfulness-based intervention (MBI), family therapy (FT), or cognitive remediation (CR), combined with SST require highly trained therapists. The vast majority of studies were not specifically designed to target negative symptoms, and most assessed negative symptoms overall, without looking for the specific effects of the intervention. Several interventions were provided over long durations, requiring a great investment in time by the patients. For example, combining CR with SST may be useful for reducing negative symptoms, particularly social withdrawal, affective flattening, and motor retardation (11, 12), or a global score of negative symptoms (13). These interventions required 32–100 h of training. One 3-year study (14) indicated that improvements in negative symptoms did not occur until the second and third years of therapy. However, a second study (15, 16) indicated improvements in negative symptoms at 12 and 24 months. These comprehensive packages are promising but involve relatively long interventions and call for a broad range of therapeutic techniques. Except SST, very few interventions are provided as group therapy. Group interventions are particularly important since they are more commonly used in community psychiatry settings, where most patients receive treatment services.

### Experimental Objectives

Recent literature has distinguished the negative symptoms associated with a diminished capacity to experience (apathy, anhedonia) from those who were associated with a limited capacity for expression (emotional blunting, alogia) (17–20). The apathy–anhedonia syndrome tends to be associated with a poorer prognosis than the symptoms related to diminished expression, suggesting that it is the more severe facet of the psychopathology (19). This syndrome is also related to the duration of the untreated psychosis, family history of schizophrenia, and the patient’s employment status during first-episode schizophrenia (21). The distinction between diminished experience and limited expression syndromes allows more specific approaches to these problems.

A symptom-specific strategy has been used in the development of specific therapeutic techniques for positive symptoms (22, 23) and led to the development of more effective interventions, such as CBT for delusions or hallucinations (24). More recently, metacognitive training (MCT), which targets associated specific cognitive biases with psychosis, has appeared effective in reducing positive symptoms (25–27). A similar strategy appears to be a good way to develop psychological interventions for negative symptoms. Recent research has also shown that more specific symptom or syndrome approaches enabled a better identification of specific psychological mechanisms. For example, the endorsement of beliefs regarding low expectations of success, and perceptions of limited personal resources, are robustly associated with negative symptoms of diminished experience (anhedonia, avolition, asociality), but are not associated with negative symptoms of diminished expressivity (flattened affect, alogia). Similarly, defeatist performance beliefs are slightly related to diminished experience, but not at all related to diminished expression (28). An impaired ability to envision the future is associated with apathy (29). These results suggest that within the syndrome of diminished capacity to experience, apathy and anhedonia may be the results of the same underlying process: that is, a diminished capacity to anticipate a particular experience or the achievement of a pleasurable goal (18), or a motivational impairment (30). This article presents a new method. It is hoped that it will open new avenues of experimental investigation for interventions to improve the diminished expression syndrome in schizophrenia. The intervention is called the positive emotions program for schizophrenia (PEPS). It comprises a program of 8 1-h sessions applied to groups of 5–10 participants. The results concerning the participants have been published elsewhere (31). This paper presents the development of the program before the pilot study with participants.

## Methods Validation

### Identification of the Component of PEPS

Anhedonia has been defined as a reduction in the ability to experience pleasure. Despite its clinical significance, research into anhedonia has produced a paradoxical set of findings, raising questions about its nature. On the one hand, using self-reported measures of trait social and physical anhedonia, individuals with schizophrenia typically report experiencing lower levels of pleasure in their daily lives than non-patients (32–35). On the other hand, in laboratory studies using emotionally evocative stimuli, individuals with schizophrenia have repeatedly reported experiencing levels of pleasant emotions similar to, or even stronger than control subjects (36–38). Germans and Kring (39) resolved this inconsistency by suggesting that patients do not anticipate that pleasurable activities will indeed be pleasurable, even though they experience pleasant emotions when presented with pleasurable stimuli. This explanation is founded on the distinction between appetitive/anticipatory pleasure (i.e., anticipating the potential pleasure of taking part in a future activity) and consummatory pleasure (i.e., the actual level of pleasure experienced directly from participating in an activity). Anticipatory pleasure is linked to motivational processes that stimulate goal-directed behaviors, whereas consummatory pleasure is associated with satiety. The Temporal Experience of Pleasure Scale (TEPS) is a trait measure of pleasure (40) that distinguishes between “momentary pleasure” and “anticipation of future pleasant activities.” A TEPS score study, comparing subjects with schizophrenia to controls, indicated that patients did not differ from controls on the consummatory scale; however, they reported significantly less anticipatory pleasure than controls (41). These results were replicated by the French version of TEPS (42). Bringing out this new way of conceptualizing anhedonia in schizophrenia permits a redefinition and calibration of the symptom complex as a target for treatment. If patients with schizophrenia show a deficit in their ability to anticipate pleasure, rather than experience pleasure, then cognitive training might well help these individuals anticipate pleasure from foreseeable, future activities. Ideally, treatment would lead to a greater ability to anticipate pleasure, and this, in turn, would lead to a meaningful increase in spontaneous daily activities. These considerations led us to explore the potential for an intervention that would train patients who complained of anhedonia, or a lack of desire to engage in activities, in the cognitive skills needed to increase their anticipatory pleasure (43). This first, exploratory pilot study included five participants with schizophrenia, presenting severe anhedonia, and stabilized on atypical antipsychotic medication. They received 10–25 h of training in anticipatory pleasure. Results showed that the patients improved on the anticipatory scale of TEPS. The patients’ daily activities were also increased according to a time budget. These preliminary data were, of course, interpreted with caution, given the small study sample, but they seemed to show a promising path toward the development of new interventions to alleviate anhedonia in schizophrenia.

Further emotional deficits may be present in schizophrenia (44) and should be taken into account in the development of new interventions (45, 46). Strauss (46) suggested maximizing positive emotional experiences by using techniques developed in the field of affective science (47, 48) to increase the frequency and duration of positive emotional experiences. Five techniques have been found to specifically and reliably increase the frequency, intensity, and duration of positive emotions, including anticipating the enjoyment. The others were behavioral display (expressing emotions via non-verbal behaviors), being “in the moment” (directing controlled attention toward positive experiences when they occur-savoring), communicating and celebrating positive experiences with others, and recalling previously pleasurable events. Patients reported lower levels of pleasure in savoring past, present, and future events than did normal controls, and stated that they had low expectations of their self-efficacy (49). Individuals with schizophrenia also manifested a lesser ability to maintain positive emotions (50–52). Even though observable, outward signs of emotional expression were lessened in schizophrenia, studies indicated that sufferers continued to display very subtle facial muscle movements (as measured by electromyogram) similar to, and in accordance with, their responses (53). Finally, to the best of our knowledge, it appears that communicating and celebrating positive events with others has not been studied in schizophrenia patients. However, one study showed that impaired perspective-taking – a component of cognitive empathy – was associated with functional capacity and community functioning, even after taking into account the influences of neurocognitive deficits and psychopathology (54).

With this as a background, Jérôme Favrod and Alexandra Nguyen conceived an intervention, which they named the “positive emotions program for schizophrenia,” to reduce anhedonia and apathy. The program teaches skills to help overcome defeatist thinking (55, 56) and to increase the anticipation and maintenance of positive emotions (44, 45). PEPS involves eight 1-h group sessions, administered using visual and audio materials as part of a PowerPoint presentation of slides projected onto a screen.

### Beta-Testing Procedure

During its development, PEPS, as well as the sensitivity of self-reporting instruments, was beta-tested on volunteer health-care professionals in order to improve its efficacy on anticipatory and consummatory pleasure, as well as savoring. Four 1-day training sessions of 7 h of PEPS were beta-tested on four different mixed groups of health-care professionals. At the end of each session, oral feedback and advice were collected, as were pre- and posttest assessments. Sessions 1–3 were conducted between February and March 2014 using version 1.0 of PEPS. Session 4 was conducted in March 2015 using the improved version 1.1 of PEPS, developed on the basis of the results of the previous sessions.

### Participants

Participants were health-care professionals, including psychiatrists, psychologists, nurses, occupational therapists, and social workers, interested in participating in the program’s development. No participant followed more than one session. Participation was anonymous, and the subjects gave only their sex and age. The clinical studies with PEPS have the agreement of the Vaud Cantonal Ethics Commission on Human Research (127/14 and 446/15).

### Instruments

The following measurement instruments were used:

• *The Savoring Belief Inventory (SBI)* is a self-reported scale for measuring beliefs about one’s capacity for savoring things. The scale has 24 items, including a positive scale (12 items) and a negative scale (12 items). The inventory has good validity and a high test–re-test reliability (57). It measures a person’s thinking regarding their capacity to savor positive experiences in terms of past experiences, current experiences, and future anticipation. The total SBI score is used.
• *The TEPS* contains 18 items included in two subscales: anticipatory pleasure (10 items) and consummatory pleasure (8 items) (58). Items targeting anticipatory pleasure reflect the pleasure felt when anticipating a positive or pleasant stimulus. Items measuring consummatory pleasure refer to the direct and immediate pleasure experienced upon exposure to a stimulus. Items can be general or specific. Responses to items fall on a six-point Likert scale from 1 (very false for me) to 6 (very true for me). This scale has been validated in French (42). The total anticipatory and consummatory scores of TEPS are used.
• *The Anticipatory and Consummatory Interpersonal Pleasure Scale (ACIPS)* (59, 60) is designed to assess one’s ability to experience pleasure in the interpersonal domain. It is a 17-item self-reported questionnaire consisting of 7 anticipatory and 10 consummatory items. ACIPS is scored on a six-point Likert scale, ranging from 1 (very false for me) to 6 (very true for me). The format is therefore quite similar to that of TEPS. The difference between the two scales lies mainly in terms of the items’ content. TEPS focuses on personal pleasure and ACIPS on interpersonal pleasure. The total anticipatory and consummatory scores of ACIPS are used.

### Data Analysis

Two-tailed paired sample *t*-tests of pre- and posttest results were calculated using IBM SPSS Statistics Version 22. Cohen’s *d* effect sizes were calculated for within-subjects in correcting for dependence among means. Formula 8 from Morris and DeShon (61) was also used.

## Results

### Results of the Beta Tests

Table 1 shows that, in session 1, the 21 participants improved on their scores significantly and clinically on the anticipatory and consummatory scales of TEPS, but not on ACIPS. Session 2 tested PEPS using the SBI scale, and scores improved clinically and significantly for the 16 participants. Session 3 replicated the previous results of ACIPS and the SBI with 27 participants. Since version 1.0 did not improve interpersonal pleasure scores as measured by ACIPS, version 1.1 was upgraded to put more emphasis on this factor. Version 1.1 includes meditations focusing on caring for others and exercises involving interpersonal pleasure. Version 1.1 was beta-tested in session 4. The 28 participants in session 4 improved their scores for both TEPS and ACIPS. No adverse effects were observed or reported during the four sessions with a total 92 different participants.

**Table 1:** **Results of the field tests with health-care professionals**.

Session	*N*	Age (SD)	Sex F/M	Scales	Pretest (SD)	Posttest (SD)	*r*	*t*	df	Two-tailed *p*	Cohen’s *d*
1	21	32.4 (8.3)	17/4	TEPS anticipatory	45.8 (7.1)	48.3 (6.8)	0.86	−3.2	20	0.005	−0.68
				TEPS consummatory	38.4 (4.8)	40.4 (4.8)	0.82	−3.1	20	0.006	−0.69
				ACIPS anticipatory	31.4 (3.6)	31.8 (4.3)	0.86	−0.9	20	0.37	−0.19
				ACIPS consummatory	51.9 (5.7)	51.7 (5.8)	0.87	0.3	20	0.71	−0.07
2	16	36.3 (8.6)	9/7	SBI total	41.4 (15.5)	47.3 (13.0)	0.93	−4.0	15	0.001	−1.12
3	27	38.0 (11.2)	21/6	ACIPS anticipatory	32.4 (3.5)	32.0 (6.8)	0.28	0.3	26	0.73	0.07
				ACIPS consummatory	52.7 (5.3)	51.9 (11.5)	0.48	0.4	26	0.69	0.09
				SBI total	36.0 (18.1)	46.0 (17.7)	0.82	−4.1	26	<0.001	−0.93
4	28	36.5 (10.9)	23/5	TEPS anticipatory	42.8 (8.1)	46.8 (5.5)	0.77	−4.2	27	<0.001	−0.90
				TEPS consummatory	38.8 (6.6)	40.9 (6.4)	0.78	−4.0	27	<0.001	−0.49
				ACIPS anticipatory	31.6 (4.9)	33.0 (5.2)	0.78	−2.3	27	0.03	−0.42
				ACIPS consummatory	49.4 (8.0)	52.8 (5.2)	0.91	−3.5	27	0.002	−0.30

*TEPS, Temporal Experience of Pleasure Scale; SBI, Savoring Belief Inventory; ACIPS, Anticipatory and Consummatory Interpersonal Pleasure Scale*.

### Detailed Description of the Final Version of PEPS

The pedagogical concept underpinning PEPS was designed according to Kolb and Kolb’s model (62) of experiential learning. This model sees the learning process as the transformation of an experience into personal knowledge. The sequential organization of the learning activity starts with the learner experiencing something (the concrete experience phase). This is followed by a stage of distancing oneself from the experience through a period of observation and reflection that seeks to give the experience meaning (the reflective observation phase). Distancing oneself from the experience broadens the learner’s understanding, generalizing, and developing concepts through more abstract thought (the abstract conceptualization phase). The learner then initiates an experimental approach to validate the newly acquired knowledge through reality tests (the active experimentation phase). This model’s major contribution is its dynamic conception of learning, seen as “a process, not in terms of results” (63). The model claims to provide a supportive environment for all learners since it is based on adults’ different learning strategies and styles, all of which can be activated through the four phases. Therefore, Kolb’s model is relevant to a therapeutic program insofar as its design corresponds to a sequential logic – alternating phases of experience and reflection. The logo at the top-left of the each slide is a reminder to group leaders as to which phase the session is in.

The program uses a collaborative, egalitarian approach. Group facilitators participate in sessions just as the participants do, by doing the exercises, sharing their experiences, and carrying out the given tasks. Group facilitators receive a day’s training before leading a group themselves and are supervised during two 1-h periods during the program.

Each session includes a number of the following steps. Part 1 begins with a welcome, followed by a 5-min relaxation–meditation exercise. In part 2, group leaders go over the homework task given during the previous session. Part 3 involves an exercise in challenging specific defeatist thoughts, which are presented using the program’s two fictitious heroes – Jill and Jack. Jill, for example, expresses such defeatist thinking as “I can’t relax; I’m useless.” The participant’s role is to challenge her belief, initially by assigning different reasons to why Jill has difficulty relaxing. They learn to find reasons that might be linked not only to the program’s heroine but also to other people or her environment. They subsequently try to develop an alternative, more positive way of thinking.

The following slides show how the exercise appears to the participants. This first slide presents a defeatist belief.


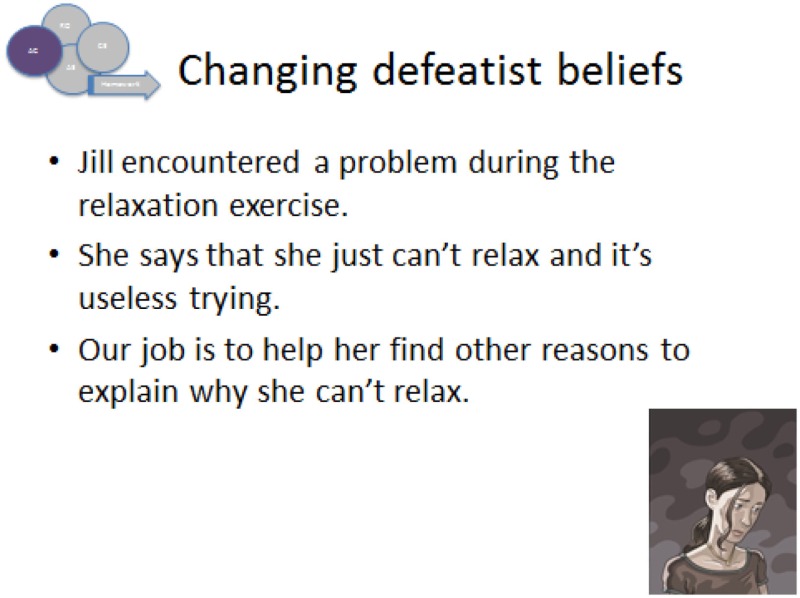


The next slide presents questions to find a more balanced set of reasons to explain Jill’s belief that she is unable to relax. Since defeatist beliefs are often linked to the internal attribution of failures, participants are asked to find a set of different reasons to explain why Jill was not able to relax. These questions are asked by the group leaders to the participants. Each of the eight sessions of PEPS will address a defeatist belief using the same methods in order to facilitate patients’ understanding of how the method works.


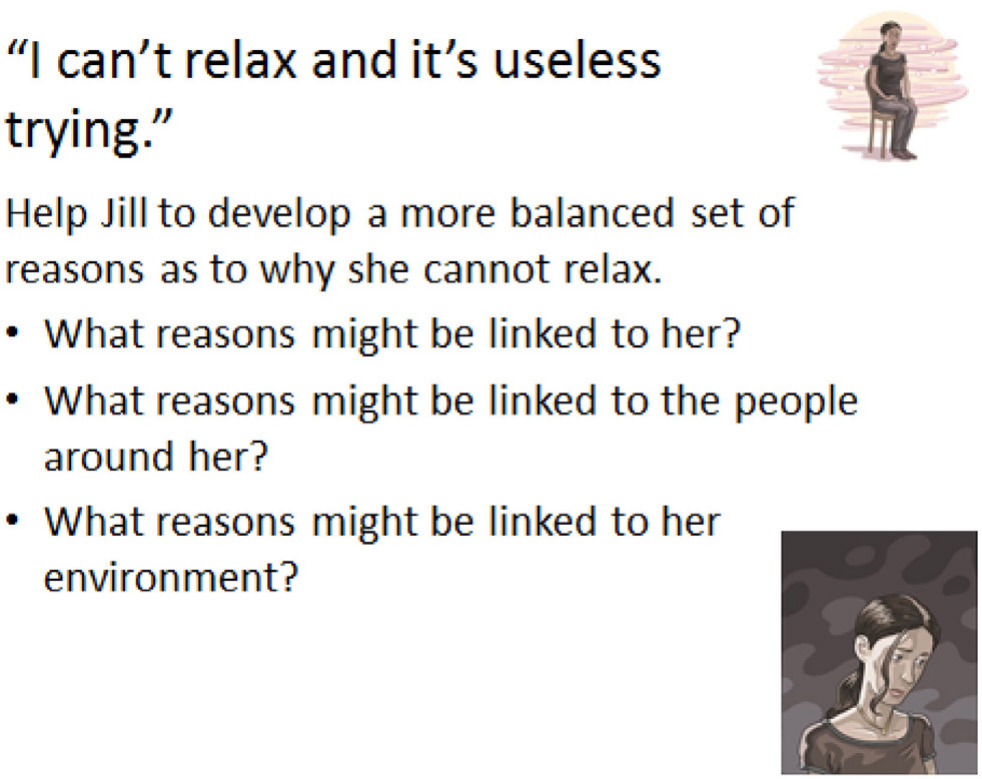


The third slide only appears after the participants have given answers to slide 2. Other suggestions are also given to complement or confirm the work they did for the previous slide.


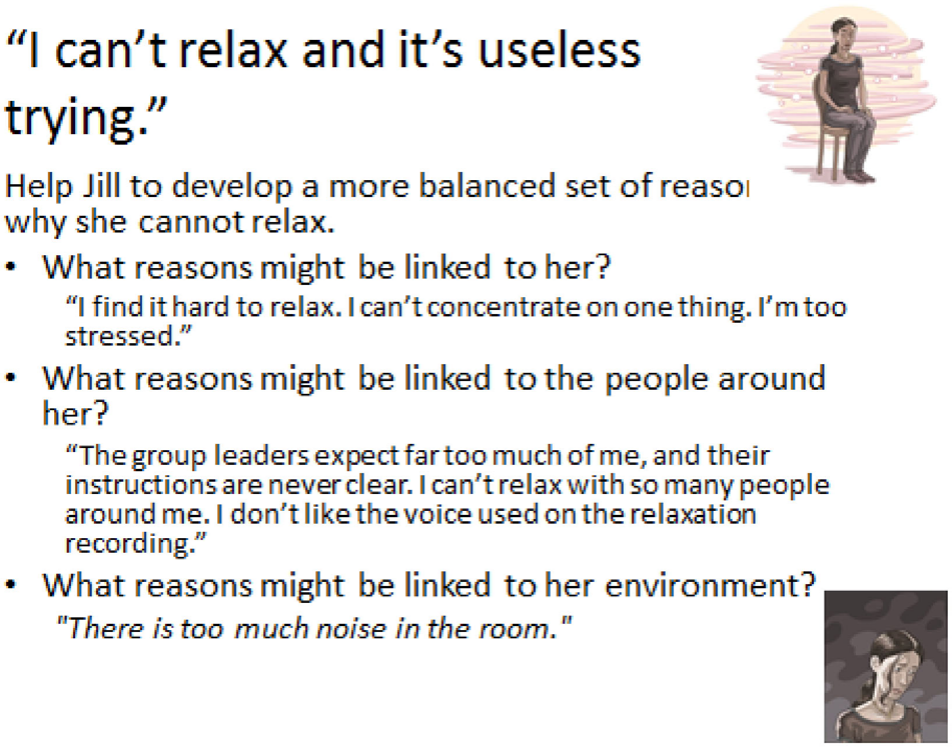


The fourth slide asks the participants to give an alternative to Jill’s conclusion and defeatist belief that she is useless.


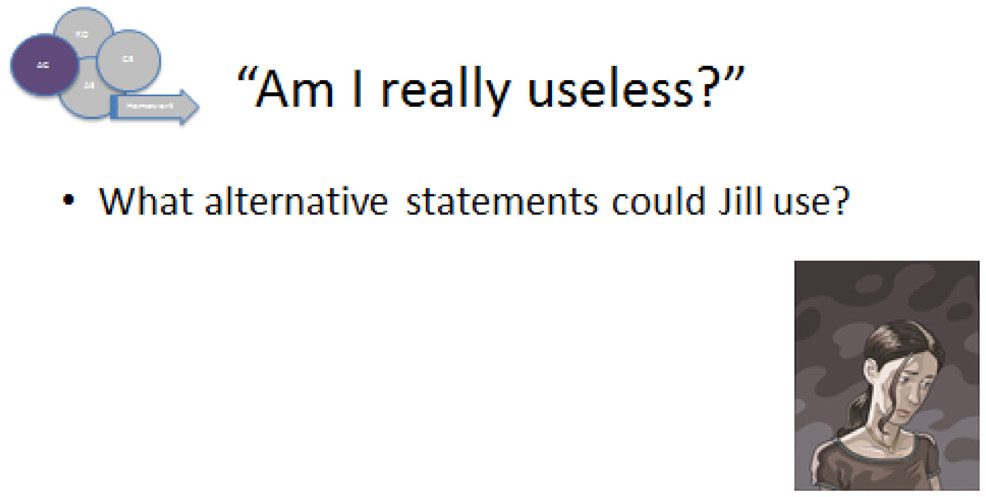


The final slide in this sequence about changing defeatist beliefs gives suggestions to complement or confirm the work the participants did for the previous slide.


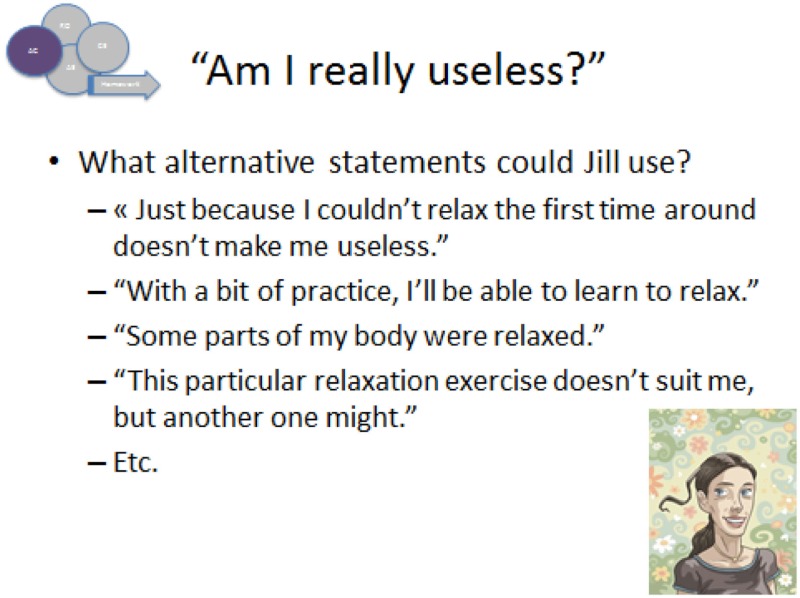


Subsequently, and according to the session’s theme, participants learn and practice a new skill to improve their anticipation or maintenance of pleasure. The session ends with group leaders setting the homework task that the participants must accomplish for the next session.

The skills taught include savoring a pleasant experience, expressing emotions by increasing behavioral expression, making the most of or capitalizing on positive moments, and anticipating pleasant moments in the future. Savoring a pleasant experience involves becoming aware of that pleasure or of the positive emotions the participant feels at a given moment (47). For example, participants are asked to look at a picture of pleasant countryside or listen to soothing music, and hence become aware of the pleasurable experience of doing this and thus appreciate it. Increasing behavioral expression of emotions involves using facial expressions or gestures to accompany that positive emotion. The participants are asked to imitate pictures of actors expressing a positive emotion and to become aware of the sensations this produces. The next slide introduces the exercise.


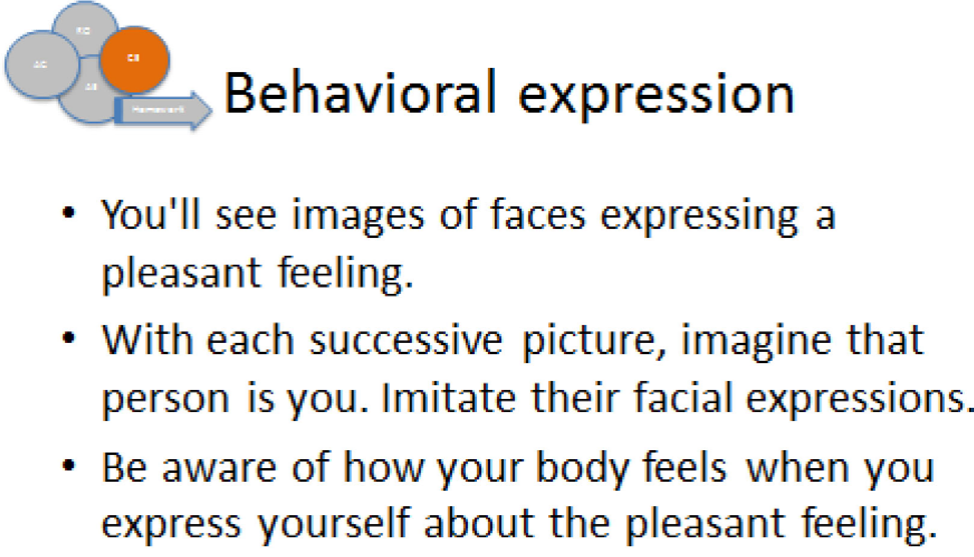


The next slide is an example of a picture to imitate.


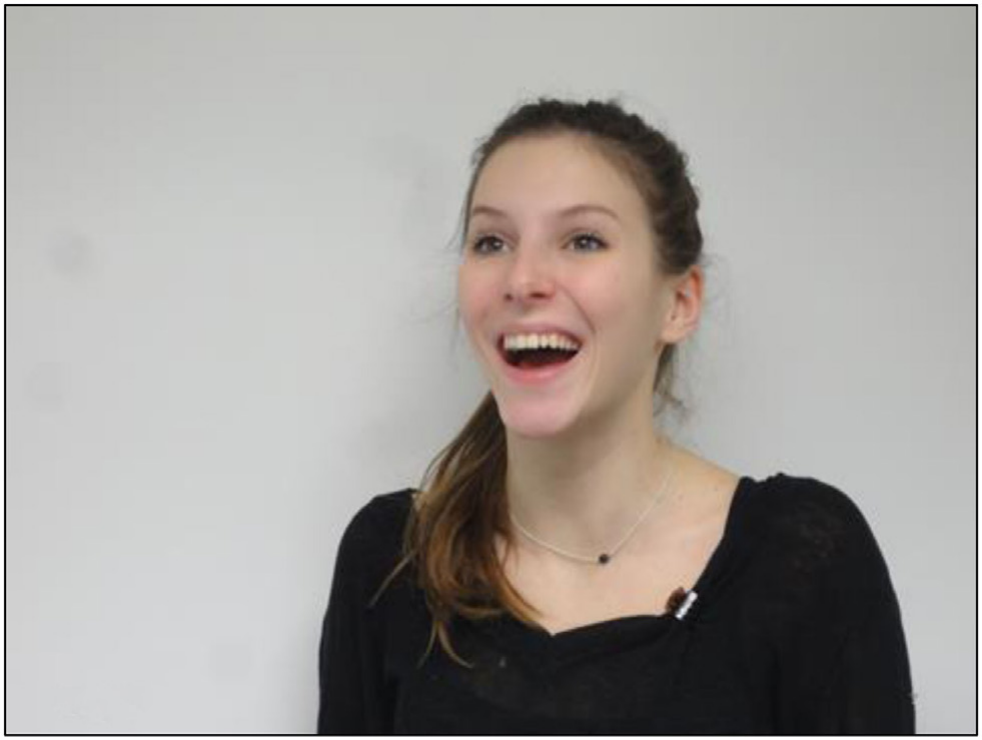


The following slide allows participants to think about the exercise. Group leaders will help each participant to share their feelings during the exercise. The group leaders will also describe what they have felt, in order to help demonstrate ways of sharing experiences and guide participants in self-observation.


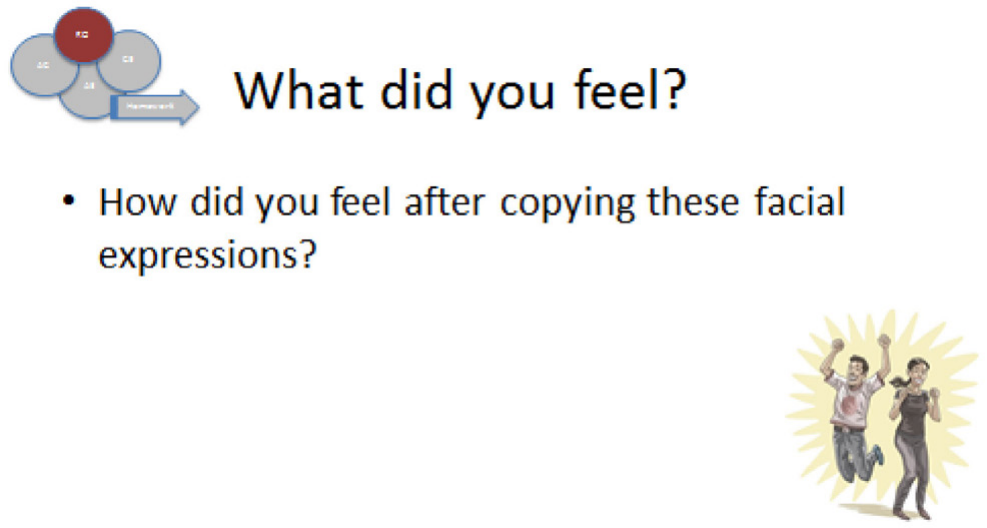


Making the most of positive moments entails communicating and celebrating positive events with others. For example, participants are asked to describe positive events to one another through role playing. The next slides show a complete sequence of training with its experiential learning steps: concrete experience, reflective observation, abstract conceptualization, and active experimentation. During the concrete experience phase, participants experience the skill they are to be taught. During reflective observation phase, they consciously express and formulate the ingredients of that experience. During abstract conceptualization phase, the participants receive theoretical information about the skill they are being taught. Finally, during active experimentation phase, they practice the skill in their natural environment as homework. The homework is reviewed at the beginning of the next session.


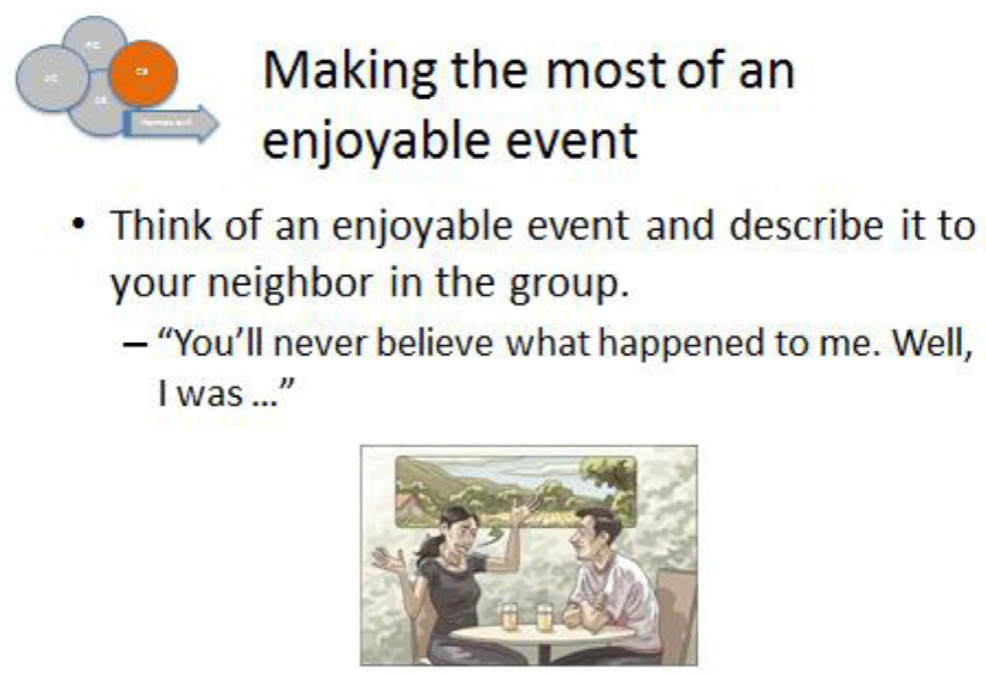



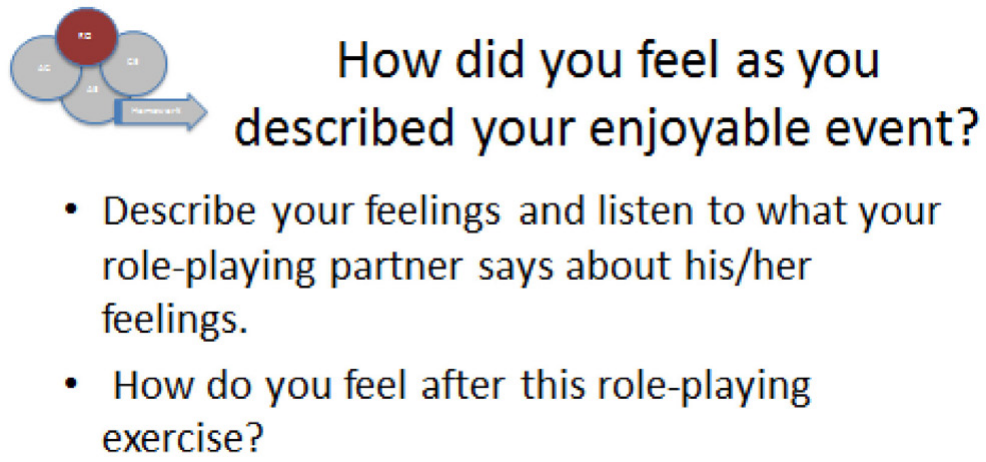



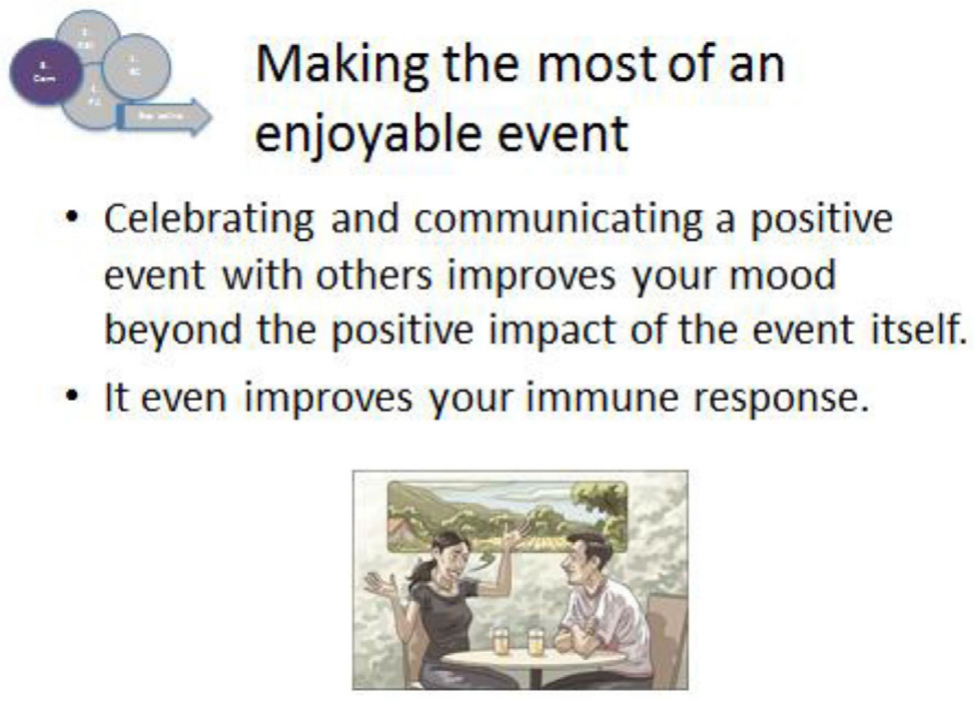



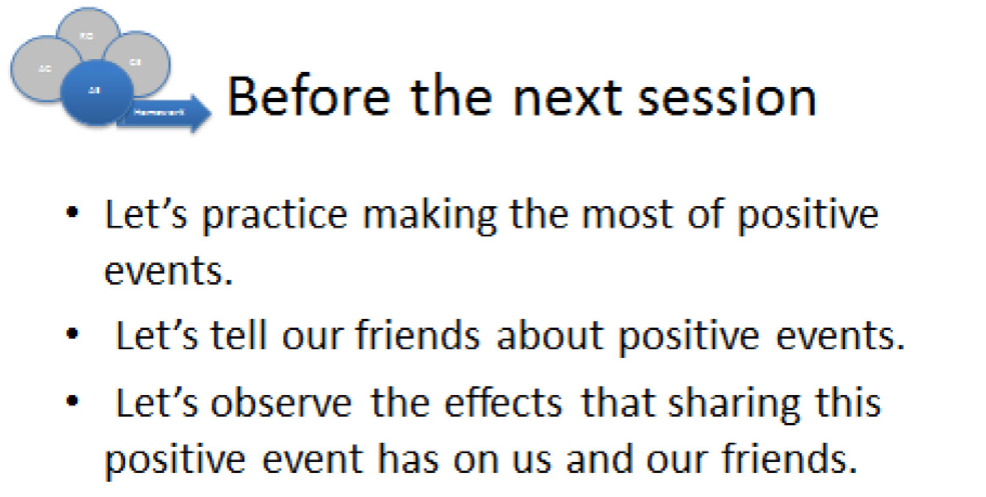


Anticipating pleasant moments involves imagining the sensations produced by positive future events. This strategy is meant to guide the participants through different positive feelings and emotions. It can engage their senses, for example, by imagining they are eating a smooth, shiny, crunchy, tasty, sweet-smelling fruit, or by anticipating the emotion produced and the physical sensations experienced upon the completion of a pleasurable physical or social activity. A simple homework task is assigned to be done between each session. For example, this could be choosing an image or an object that provokes a positive emotion or feeling in the participant, who must then bring it back and describe it to the group. The original French version of PEPS can be downloaded for free at http://www.seretablir.net/outils-interventions/peps/.

## Discussion

### Key Findings

Drug and psychological treatments for the negative symptoms of schizophrenia have shown poor clinical efficacy. Furthermore, they require lengthy therapeutic interventions involving highly skilled professionals. This paper presents a specific, short, easy to use, group-based intervention to improve pleasure, and motivation in schizophrenia. A more targeted syndrome approach was grafted to a model of the advancement of psychological therapies for delusions. The program targets apathy and anhedonia as they can be combined into a single reduced-experience syndrome. The program was built with regard to the specific deficits described in the literature on pleasure and motivation. The program was developed by beta-testing on health-care professionals and was improved according to the results of earlier tests. A pilot study was conducted with participants who met the ICD-10 criteria for schizophrenia or schizoaffective disorders (31). Thirty-one participants completed the program; those who dropped out did not differ significantly from completers. Participation in the program was accompanied by statistically significant reductions in the total scores for avolition–apathy and anhedonia–asociality on the Scale for the Assessment of Negative Symptoms, with moderate effect sizes. Furthermore, there was a statistically significant reduction of depression on the CDSS, with a large effect size. Emotional blunting and alogia remained stable during the intervention. The original program in French can be downloaded for free on the Internet. It was designed to be easy to use and applicable in group sessions so as to meet the needs of community care psychiatry.

### Potential Shortcomings and Limitations

Positive emotions program for schizophrenia was designed using a recovery-oriented approach rather than a deficit-centered approach. The deficits of pleasure, and above all of motivation, may be related to factors that were not selected as targets in the program. This could be mainly because the authors were looking for a rapid, targeted intervention on the diminished experience syndrome. For example, apathy is associated with poor performance on executive tests (64–66). CR has been shown to improve executive functioning, but although CR yields lasting effects on global cognition and functioning, its influence on symptom effects is small and disappears over time (67). However, a recent study indicated that anticipation abnormalities are associated rather to negative beliefs about potentially rewarding social situations than neurocognitive deficits (68). Motivation may also be affected by medication; for example, the D2 antagonistic effect of antipsychotic agents reduces anticipation of a monetary reward (69). PEPS does not include medication in its intervention.

The use of health-care professionals in the beta tests for developing the program may be questionable since they were not representative groups of people with schizophrenia. The high female-to-male ratio of the beta test samples did not fit with the high male-to-female ratio of patients with enduring schizophrenia (70). However, these professionals were easy to reach out to, familiar with the problems surrounding schizophrenia, and able to give knowledgeable feedback on PEPS exercises. However, this familiarity with schizophrenia may also be a bias against innovation. The developers were well aware of these risks. The main reasons for the beta tests were to quickly evaluate the intervention’s feasibility, its impact on the self-reporting scales selected, and the program’s safety before the clinical phase in the development of PEPS.

### Future Directions

The findings presented here indicate that PEPS is indeed a feasible intervention, and it was associated with an apparently specific reduction of anhedonia and apathy. However, these findings are limited by the absence of a control group and the fact that the rater was not blind to the treatment objectives. A randomized controlled study with blind raters is needed to assess more correctly the efficacy of PEPS.

## Author Contributions

AN and JF, in equal measure, conceptualized this research and PEPS, acquired, analyzed, and interpreted the data, and drafted the first version of the manuscript. LF, IM, PG, and CB gave a substantial contribution to the analysis and interpretation of data and critically revised the article for important intellectual content. All the authors approved the final version for publication. All the authors agree to be accountable for all aspects of the work by ensuring that any questions related to its accuracy or integrity can be appropriately investigated and resolved.

## Conflict of Interest Statement

The authors declare that this research was conducted in the absence of any commercial or financial relationships that could be construed as a potential conflict of interest.
